# First - line, non - cryopreserved autologous stem cell transplant for poor - risk germ - cell tumors: Experience in a developing country

**DOI:** 10.1590/S1677-5538.IBJU.2017.0562

**Published:** 2019

**Authors:** Eucario Leon-Rodriguez, Monica M. Rivera-Franco, Dennis Lacayo-Leñero, Andrea Campos-Castro, Monica I. Meneses-Medina

**Affiliations:** 1 Stem Cell Transplantation Program, Department of Hematology and Oncology, Instituto Nacional de Ciencias Medicas y Nutricion Salvador Zubiran, Mexico City, Mexico;; 2 Stem Cell Transplantation Program,Hematology Section, Department of Hematology and Oncology, Instituto Nacional de Ciencias Medicas y Nutricion Salvador Zubiran, Mexico City, Mexico;; 3 Stem Cell Transplantation Program, Oncology Section, Department of Hematology and Oncology, Instituto Nacional de Ciencias Medicas y Nutricion Salvador Zubiran Mexico City, Mexico.

**Keywords:** Stem Cell Transplantation, Neoplasms, Cryopreservation

## Abstract

**Purpose:**

The current first - line treatment for non - seminomatous germ cell tumor (NSGCT) consists of four cycles of cisplatin, etoposide, and bleomycin (BEP), which results in 5 - year overall survival < 60% in patients with poor - risk features. Autologous hematopoietic stem cell transplantation (auto - HSCT) as a method for overcoming high toxicity after high dose chemotherapy (HDC) has been explored in different solid tumors, but has remained standard practice only for NSGCT. Our objective was to describe outcomes of patients with poor - risk NSGCT who underwent first - line autologous HSCT in a tertiary center in Mexico.

**Patients and Methods:**

Twenty nine consecutive patients with NSGCT who received first - line, non - cryopreserved autologous HSCT at the National Institute of Medical Sciences and Nutrition Salvador Zubiran in Mexico City, Mexico, from November 1998 to June 2016, were retrospectively analyzed.

**Results:**

The median age at transplantation was 23 (15 – 39) years. Most patients (n = 18, 62%) had testicular primary tumor, and 23 had metastases (79%). Complete response after auto - HSCT was observed in 45%. Non - relapse mortality was 0. Five - year relapse / progression free and overall survival were 67% and 69%, respectively.

**Conclusions:**

This small single limited - resource institution study demonstrated that patients with poor - risk NSGCT are curable by first - line HDC plus autologous HSCT and that this procedure is feasible and affordable to perform using non - cryopreserved hematopoietic stem cells.

## INTRODUCTION

Testicular cancer accounts for 1.5% and 5% of male and urological tumors, respectively. Annually, three to six new cases occur per 100,000 men in the United States and up to 5% are bilateral. In Mexico, approximately 1.742 new cases were diagnosed in 2012 (1) and according to the Mexican Malignant Tumor Histopathological Register (2001), testicular cancer is the most curable solid tumor and the most frequent urological tumor in working - age men (2). The vast majority of non - seminomatous germ cell tumor (NSGCT) arise in the testicles with ~5% occurring outside of the gonads (3). Cisplatin - based chemotherapy can cure patients with disseminated NSGCT, even with visceral metastases, highly elevated serum tumor markers, and other adverse features, however results are better in patients of favorable risk group. The current first - line treatment consists of four cycles of cisplatin, etoposide, and bleomycin (BEP) (3), which results in 5 - year overall survival (OS) rates of less than 60% in patients with advanced NSGCT classified with poor - risk features according to the International Germ Cell Cancer Collaborative Group (IGCCCG) Classification (4-6).

A solution for overcoming resistance mechanisms to chemotherapy in patients with malignant diseases has been to increase the dose of cytotoxic drugs. However, it was not possible to administer higher doses due to the high toxicity and potentially lethal toxicity on bone marrow. Therefore, autologous hematopoietic stem cell transplantation (auto - HSCT) as a method for overcoming this high toxicity after chemotherapy has been explored in different solid tumors, but has remained standard practice only for NSGCT. In fact, this procedure (high - dose chemotherapy (HDC) followed by auto - HSCT) has been studied in both first - line and salvage settings in NSGCT. The relative tolerability and improvement in relapse / progression free survival (PFS) and overall survival (OS) compared with historical controls treated with conventional - dose programs suggested that this was a promising approach (7-10). Nonetheless, so far, there is no clear evidence that HDC plus auto - HSCT given as first - line therapy increase survival in patients with poor-risk NSGCT and the unresolved question as to what constitutes optimal therapy for these patients remains. On the other hand, while most patients with metastatic NSGCT are cured with first - line chemotherapy, the cure rate for those that relapse after first - line chemotherapy for metastatic disease is much lower.

The aim of this study was to describe outcomes of patients with poor - risk NSGCT who underwent HDC followed by auto - HSCT in a National Health Institute in Mexico.

## PATIENTS AND METHODS

Consecutive patients with poor - risk NSGCT treated at a National Institute of Mexico, National Institute of Medical Sciences and Nutrition Salvador Zubiran in Mexico City, from November 1998 to June 2016, were retrospectively analyzed. Thirty seven patients were identified (8 refractory / relapsed), but only 29 received an auto - HSCT as first - line treatment. Clinical and pathological characteristics such as age, sex, histology, disease extent, tumor markers, and responses were included. Follow - ups were done in the out - patient clinic. Diagnosis was made by the histopathological report from the ultrasound or CT - guided fine needle aspiration biopsy (mediastinal and retroperitoneal tumors), or from the inguinal orchiectomy (testicular tumors) plus serum markers such as alpha - fetoprotein (AFP), lactate dehydrogenase (LDH), and human chorionic gonadotropin (β - hCG). Second or third transplantations were excluded. The dataset used for this study derived from the Transplantation Program records, archived in the Department of Hematology and Oncology, containing all the information of the in - patient procedure. Also, hospital official medical records, and electronic records, including imaging and pathology, were reviewed. All patients signed an informed consent before undergoing HSCT. The Institutional Review Board approved the usage of patient information for this study.

### Hematopoietic Stem Cell Transplantation

All patients were admitted one day prior the administering the conditioning regimen, and were placed in rooms with only high - efficiency particulate air (HEPA) filters during their in - patient stay. Autologous hematopoietic stem cells (HSCs) were collected by peripheral blood stem cell (PBSC) apheresis. Patients received granulocyte - colony stimulating factor (G - CSF) (10 μg / kg / day) 5 days prior this procedure. Conditioning regimen was etoposide (1200 mg / m2, IV) and carboplatin (1400 mg / m2, IV) administered during three consecutive days. Previously collected CD34 + cells were infused 5 – 6 days after harvesting, thus cryopreservation was not performed. Blood products and nutritional support were provided according to institutional guidelines. All blood products were filtered and radiated. G - CSF was administered on day + 5 until hematopoietic recovery (approximately 1 week). No physical or occupational therapy was given. Antimicrobial prophylaxis consisted of ciprofloxacin (500 mg / 12 hours, oral) and acyclovir (250 mg / 8 hours, IV), administered starting the conditioning regimen. Antifungal prophylaxis included fluconazole (400 mg / daily, IV) starting day 1 of conditioning regimen.

### Outpatient follow-up

Patients were discharged when engraftment occurred and in the absence of infections or complications. Out - patient visits took place twice a month during the first two months. Requested labs included complete blood count (CBC) and liver panel. No medications were prescribed. Patients in whom markers normalized, but who showed evidence of residual tumor mass, underwent debulking surgery.

### Endpoints and definitions

Poor - risk NSGCT were defined by one or more of the following criteria: primary mediastinal NSGCT or by testicular / retroperitoneal primary NSGCT with non - pulmonary visceral metastasis, AFP > 10 000 ng / mL or β - hCG > 50 000 mIU / mL, or LDH > 10 × upper limit of normal (4). The following institutional laboratory references were considered: AFP 0-9 ng / mL, β - hCG 0.5 – 2.67 mUI / mL, and LDH 140-271 U / l. Morbidity post - transplant was analyzed according to the NCI Common Terminology Criteria for Adverse Events (CTCAE v4.0). Neutrophil engraftment was considered as ANC ≥ 0.5 × 10^9^ / L in the first day of three consecutive days. Platelet engraftment was defined as a platelet count ≥ 20 × 10^9^ / L, during three consecutive days without transfusions. Graft failure was defined as the inability to achieve neutrophils and platelets engraftment during 45 days post - transplant. Patients with normal levels of tumor markers and no clinical or radiological evidence of residual masses were classified as complete responders (CR) and were monitored without further therapy. Non - relapse mortality (NRM) was defined as death related to the conditioning regimen or infections during aplasia without relapse / progression and excluding causes due to underlying disease. Progression / relapse free survival (PFS) was established as the length of time from transplantation until relapse or progression of the underlying disease. Overall survival (OS) was defined as time from transplantation until death from any cause.

### Statistical analysis

Frequencies and percentiles were used to describe categorical variables. Continuous variables were described by the median and interquartile range using the frequency analysis. The OS for all patients was calculated using the Kaplan Meier estimator. Cumulative incidence estimates were calculated for other endpoints (NRM, relapse / progression) to account for competing risks. Non - relapse death was a competing risk in the estimation of relapse and relapse a competing risk for non - relapse mortality (NRM). Two - sided p - value of < 0.05 were considered significant. SPSS v. 21 (IBM, Chicago, IL) was used.

## RESULTS

Patients: Twenty nine consecutive patients diagnosed with germ cell tumor were included (Table-1). The median age at diagnosis and at transplantation were 23 (15 – 39) and 23 (17 – 39) years, respectively. All patients had an Eastern Cooperative Oncology Group (ECOG) performance status of 0. Most frequent histology was mixed germ cell tumors (n = 8, 28%), followed by malignant teratoma (n = 4, 14%). Eighteen patients (62%) had testicular primary tumor, and 23 patients had metastases (79%), most commonly in the lungs (n = 14, 61%). At diagnosis, 28 patients (97%) had positive tumor markers. According to the International Germ Cell Consensus Classification, all patients were poor - risk. Prior HSCT, 9 patients (31%) persisted with positive tumor markers. Twenty patients (69%) had residual tumor prior HSCT.

**Table 1:** Patient demographics.

Characteristic		n (%)
**Total**		29 (100)
**Age (median, range)**		
	Diagnosis	23 (15-39)
	HSCT	23 (17-39)
**Histology**		
	Embryonal carcinoma	2 (7)
	Yolk sac tumor	2 (7)
	Choriocarcinoma	3 (9)
	Malignant teratoma	4 (14)
	Mixed	8 (28)
	Other	4 (14)
	Unknown	6 (21)
**Primary tumor**		
	Testicular	18 (62)
	Mediastinal	9 (31)
	Retroperitoneal	2 (7)
**Risk stratification**		
	Poor-risk	29 (100)
**Metastases**		
	Yes	23 (79)
	No	6 (21)
**Number of disease sites**		
	1	8 (28)
	2	7 (23)
	> 3	8 (28)
	Unknown	6 (21)
**Metastatic sites**		
	Lung	14
	Retroperitoneum	10
	Mediastinum	3
	Liver	9
	Brain	1
	Cervical lymph nodes	3
**Positive tumor markers at diagnosis**		
	Yes	28 (97)
	No	1 (3)
**Tumor markers at diagnosis**		
	AFP median (range) (ng/mL)	24 (1.9-20928)
	β-hCG median (range) (mUI/mL)	395 (0-395790)
	LDH median (range) (U/l)	622 (152-14510)
**Positive tumor markers prior HSCT**		
	Yes	9 (31)
	No	20 (69)
**Tumor markers prior HSCT**		
	AFP median (range) (ng/mL)	4.4 (1.8-190)
	β-hCG median (range) (mUI/mL)	0.6 (0-1141.5)
	LDH median (range) (U/l)	152 (86-331)
**Chemotherapy regimens prior HDCT + HSCT**		
	1	18 (62)
	2	5 (17)
	> 3	2 (7)
	Unknown	4 (14)
**Response to initial standard chemotherapy**		
	CR	3 (10)
	PR	18 (62)
	Unknown	8 (28)
**Residual tumor prior HSCT**		
	Yes	14 (49)
	No	10 (34)
	Unknown	5 (17)

**AFP =** Alpha-fetoprotein; **β -hCG =** Beta subunit of human chorionic gonadotropin; **CR =** Complete response; **IPFSG =** International prognostic factors study group; **LDH** = lactate dehydrogenase; **PR =** Partial response.

Autologous HSCT: Most patients received a single chemotherapy regimen prior becoming candidates for HSCT (n = 18, 62%). Eighteen patients (62%) showed partial response to initial chemotherapy. For the HSCT procedure, all patients received etoposide and carboplatin as pre transplant conditioning regimen. The median infused CD34 + cells was 3 × 10^6^ / kg (1.3 – 10.2). G - CSF was administered to 21 patients (72%) starting with a median 4 days (0 – 14) post - transplant and during a median 8 days (0 – 17).

Conditioning regimen toxicity: Conditioning related toxicity was observed in 27 (93%) patients (35% grades III - IV). The most common affection was hepatic toxicity (n = 17, 63%) being grades III - IV in 47%. Thirteen patients (48%) developed oral mucositis (8% grades III - IV). None of the patients developed central nervous system toxicity.

Infections: Twenty four patients (83%) developed infections during the in - patient stay with a median onset of 6 days (0 – 14). Colorectal infections affected 8 patients (33%) followed by catheter in 7 (29%), neutropenic fever in 6 (25%), and other infections 13%. Pathogens were isolated in 8 patients (33%), all bacterial.

Transfusions and engraftment: Median transfused red blood cell and apheresis platelets units were 0 (0 – 4) and 2 (0 – 7), respectively. None of the patients presented refractoriness to infused platelets. Median engraftment for neutrophils and platelets were 12 (8 – 20) and 11 (7 – 21) days, respectively. Median hospitalization days were 20 (14 – 33).

**Figure 1:**
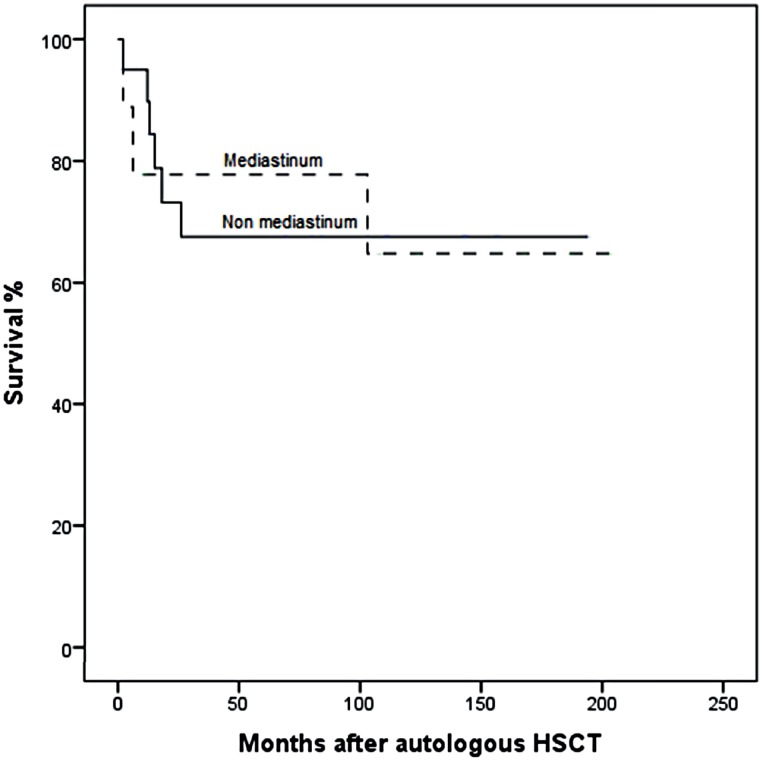
Overall survival comparing site of primary tumor (mediastinum and non - mediastinum).

**Figure 2:**
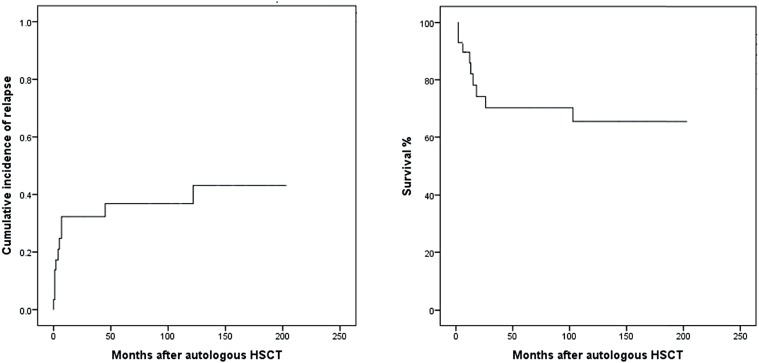
Progression and overall survival.

Response, progression and survival: Complete response was observed in 13 patients (45%). The median post - transplant tumor markers were: 159 (114 – 357) U / l, 0.32 (0 – 5432) mUI / mL, and 3.9 (0.9 – 290) ng / mL for LDH, β - hCG, and AFP, respectively. Eleven patients (38%) persisted with residual tumor after HSCT. Eleven patients (38%) relapsed/progressed after HSCT. After a median follow-up of 103 months (2 – 203), 20 patients (69%) were alive. NRM within 30 and 100 days were 0. Most frequent cause of death was relapse (n = 5, 56%) (Table-2). Five - year relapse free survival and OS were 67% and 69%, respectively. Comparing site of primary tumor (mediastinum and no mediastinum), 5 - year OS was 78 and 67%, respectively (depicted in Figure-1). Relapse and OS are shown in Figure-2.

**Table 2:** Causes of death (n=9).

Cause	n, %
**Relapse or progression**	5 (56)
**Other causes not related with HSCT**	
Infections after aplasia	1 (11)
Radiotherapy	1 (11)
Others	2 (22)

## DISCUSSION

Most patients with metastatic NSGCT are cured with standard chemotherapy: 3 – 4 cycles of BEP protocols (3, 11). However, in patients with poor - risk, cure rate is below 50%. In this group and in patients with relapsed testicular cancer, unsatisfactory performance standard chemotherapy has directed researchers to search for new forms of treatment. The rationale of using high dose chemotherapy (HDC) in chemo - sensitive cancer lead on investigators to start clinical trials with high dose chemotherapy and stem cell support (12, 13). In the 90s, the initial studies of high dose chemotherapy for patients with poor-risk in the first - line setting were completed (7, 8, 14). The first study showed that 15 of 27 patients (56%) achieved complete remission (CR), 46% were disease free, and 57% alive after a median follow-up of 31 months (8). Three years later, the same authors treated 30 patients, 16 with VIP chemotherapy, and the rest (14 patients) received HDC (carboplatin, etoposide, and cyclophosphamide (CEC)) after VIP. Patients whose tumor markers did not normalize after two cycles of chemotherapy were selected for HDC. After a median follow-up of 30 months, 50% remained progression - free. Patients treated with marker - dependent, early - intervention HDC experienced longer survival (7). In 1999, Bokemeyer et al. (14) published an analysis comparing the outcomes of patients with poor - risk metastatic NSGCT treated sequentially with standard VIP and HDC in a multi - centric study. They included patients from German group studies and patients treated in two studies from Indiana University, with BEP or VIP conventional chemotherapy. HDC group included 147 patients, while 309 patients were in the conventional chemotherapy group. Patients treated with HDC had a longer PFS 75% vs. 59% (p = 0.0056) and a longer OS 82% vs. 71% (p = 0.0184) (7). After that, Schmoll et al. (10) published a phase I / IIa study where they treated poor - risk NSGCT patients with a VIP - escalated protocol (one cycle of standard VIP, followed by 3 – 4 cycles of high dose escalated VIP with auto - HSCT). Five - year PFS in this group of patients was 68%, which was longer than the historical control with standard chemotherapy.

The German Testicular Cancer Study Group (GTCSG) added paclitaxel, after the proven effectiveness of this drug in cisplatin-resistant NSGCT, to dose - escalated VIP protocol (15). Addition of paclitaxel to HDC VIP resulted in higher response rate of 79%, and five - year PFS and OS of 64% and 75%, respectively (15).

Motzer et al. (13) have published the only randomized phase III study including 219 untreated intermediate and poor - risk metastatic NSGCT patients. One group of patients was treated with standard therapy (4 cycles BEP), while the experimental group received two cycles of BEP and followed by two cycles of high dose CEC, showing benefit of HDC only in those patients with unsatisfactory tumor markers decline. One year complete remission was not different in the two groups of patients (48% vs. 52%, p = 0.53). The authors reported a 2 - year survival of 67% and a time - to - treatment failure (TTF: treatment to relapse) of 23.2 months in the group receiving HSCT. However, this study included patients with intermediate - risk features (20%) and chemotherapy doses were lower (carboplatin 600 mg / m^2^ and etoposide 600 mg / m^2^, plus cyclophosphamide 50 mg / kg) than our study. Nonetheless, our survival compares with their results (69 vs. 67%, respectively), but the median of TTF in our study has not been reached.

Other studies started the third phase, but due to poor recruiting of patients they were not fully completed (16-18). Daugaard et al. (18) reported a 2 - year failure free survival (FFS) of 58.2% after HDC using VIP regimens. Overall, the analysis of the recruited patients from HDC did not show the expected benefit in first - line treatment of metastatic NSGCT with a poor - risk.

On the other hand, several studies have used HDC showing promising response rates in refractory NSGCT (19-21). In our experience, although limited (8 patients), it seems that auto - HSCT does not have a role in first - line for platinum refractory NSGCT, but it does as salvage therapy obtaining a 5 - year PFS and OS of 50% and 67%, respectively [results not shown].

We acknowledge that the main limitations of our study include a retrospective, small cohort, and heterogeneous histological subtypes. However, this small single limited - resource institution study demonstrated that patients with poor - risk NSGCT are curable by first - line HDC plus non - cryopreserved autologous HSCT. To our knowledge, this is the first published study in a developing country and using non-cryopreserved hematopoietic stem cells. The relapse / progression free and overall survival using HCT and autologous HSCT were 67% and 69%, respectively. We emphasize that demonstrating feasibility using non - cryopreserved HSCs facilitates the widespread use of autologous HSCT in centers without this infrastructure, which is frequent at limited - resource centers in low - middle income countries, contributing to the affordability of the overall procedure. By this means, engraftment was similar to reported literature (neutrophils and platelets, 12 and 11 days, respectively) and median hospitalization was short (20 days). Toxicity has been reported to be more severe for patients treated with HDC associating this complication with mortality, especially secondary to septic shock (16-18). Grades III - IV HDC - related toxicity was present in 35% of our cohort and was not associated with mortality or further complications. Also, febrile neutropenia has been reported in > 90% of patients receiving HDC (18), compared to 25% in our study. Colorectal infections affected 33% of our cohort, compared to other studies reporting up to 59% gastrointestinal infections (17). Among our cohort, NRM at 30 and 100 days was 0. Causes of death were mostly as a consequence of relapse.

Further, our results administering HDC plus one autologous HSCT compare to studies using 2 – 4 first - line HDC plus stem cell support (auto - HSCT), which highlights accessibility in developing countries. Moreover, we have previously reported the costs of HSCT in Mexico (22), demonstrating that it is an affordable procedure (median cost of autologous HSCT 12, 155 USD), and avoiding cryopreservation allows this cost to remain low especially in limited - resource centers without facilities to freeze and store hematopoietic cells as it is expensive, complex, and requires substantial expertise.

Also, patients with non - mediastinal and mediastinal NSGCT had similar outcomes, supporting the use of HSCT plus auto - HSCT as first - line treatment in the latter, but larger prospective studies are needed.

In conclusion, our approach (HDC plus non - cryopreserved auto - HSCT) as first - line treatment in poor - risk NSGCT is a feasible procedure in low and middle income countries due to similar results compared to published prospective studies in developed countries.

## References

[1] Ferlay J, Soerjomataram I, Ervik M (2013). GLOBOCAN 2012 v1.0, Cancer Incidence and Mortality Worldwide: IARC CancerBase No. 11. Lyon.

[2] Dirección General de Epidemiología (2001). Registro Histopatológico de Neoplasias.

[3] Oldenburg J, Fosså SD, Nuver J, Heidenreich A, Schmoll HJ, Bokemeyer C (2013). Testicular seminoma and non-seminoma: ESMO Clinical Practice Guidelines for diagnosis, treatment and follow-up. Ann Oncol..

[4] (1997). International Germ Cell Consensus Classification: a prognostic factor-based staging system for metastatic germ cell cancers. International Germ Cell Cancer Collaborative Group. J Clin Oncol..

[5] Williams SD, Birch R, Einhorn LH, Irwin L, Greco FA, Loehrer PJ (1987). Treatment of disseminated germ-cell tumors with cisplatin, bleomycin, and either vinblastine or etoposide. N Engl J Med..

[6] Hinton S, Catalano PJ, Einhorn LH, Nichols CR, David Crawford E, Vogelzang N (2003). Cisplatin, etoposide and either bleomycin or ifosfamide in the treatment of disseminated germ cell tumors: final analysis of an intergroup trial. Cancer..

[7] Motzer RJ, Mazumdar M, Bajorin DF, Bosl GJ, Lyn P, Vlamis V (1997). High-dose carboplatin, etoposide, and cyclophosphamide with autologous bone marrow transplantation in first-line therapy for patients with poor-risk germ cell tumors. J Clin Oncol..

[8] Motzer RJ, Mazumdar M, Gulati SC, Bajorin DF, Lyn P, Vlamis V (1993). Phase II trial of high-dose carboplatin and etoposide with autologous bone marrow transplantation in first-line therapy for patients with poor-risk germ cell tumors. J Natl Cancer Inst..

[9] Decatris MP, Wilkinson PM, Welch RS, Metzner M, Morgenstern GR, Dougall M (2000). High-dose chemotherapy and autologous haematopoietic support in poor risk non-seminomatous germ-cell tumours: an effective first-line therapy with minimal toxicity. Ann Oncol..

[10] Schmoll HJ, Kollmannsberger C, Metzner B, Hartmann JT, Schleucher N, Schöffski P (2003). Long-term results of first-line sequential high-dose etoposide, ifosfamide, and cisplatin chemotherapy plus autologous stem cell support for patients with advanced metastatic germ cell cancer: an extended phase I/II study of the German Testicular Cancer Study Group. J Clin Oncol..

[11] Albers P, Albrecht W, Algaba F, Bokemeyer C, Cohn-Cedermark G, Fizazi K (2011). EAU guidelines on testicular cancer: 2011 update. Eur Urol..

[12] Necchi A, Lanza F, Rosti G, Martino M, Farè E, Pedrazzoli P (2015). High-dose chemotherapy for germ cell tumors: do we have a model?. Expert Opin Biol Ther..

[13] Motzer RJ, Nichols CJ, Margolin KA, Bacik J, Richardson PG, Vogelzang NJ (2007). Phase III randomized trial of conventional-dose chemotherapy with or without high-dose chemotherapy and autologous hematopoietic stem-cell rescue as first-line treatment for patients with poor-prognosis metastatic germ cell tumors. J Clin Oncol..

[14] Bokemeyer C, Kollmannsberger C, Meisner C, Harstrick A, Beyer J, Metzner B (1999). First-line high-dose chemotherapy compared with standard-dose PEB/VIP chemotherapy in patients with advanced germ cell tumors: A multivariate and matched-pair analysis. J Clin Oncol..

[15] Hartmann JT, Gauler T, Metzner B, Gerl A, Casper J, Rick O (2007). Phase I/II study of sequential dose-intensified ifosfamide, cisplatin, and etoposide plus paclitaxel as induction chemotherapy for poor prognosis germ cell tumors by the German Testicular Cancer Study Group. J Clin Oncol..

[16] Di Nicola M, Necchi A, Nicolai N (2009). High-dose sequential chemotherapy versus conventional-dose chemotherapy as first-line treatment for advanced poor prognosis germ-cell tumors: a multicenter Phase III Italian trial. EJC Supplements..

[17] Necchi A, Mariani L, Di Nicola M, Lo Vullo S, Nicolai N, Giannatempo P (2015). High-dose sequential chemotherapy (HDS) versus PEB chemotherapy as first-line treatment of patients with poor prognosis germ-cell tumors: mature results of an Italian randomized phase II study. Ann Oncol..

[18] Daugaard G, Skoneczna I, Aass N, De Wit R, De Santis M, Dumez H (2011). A randomized phase III study comparing standard dose BEP with sequential high-dose cisplatin, etoposide, and ifosfamide (VIP) plus stem-cell support in males with poor-prognosis germ-cell cancer. An intergroup study of EORTC, GTCSG, and Grupo Germinal (EORTC 30974). Ann Oncol..

[19] Nichols CR, Tricot G, Williams SD, van Besien K, Loehrer PJ, Roth BJ (1989). Dose-intensive chemotherapy in refractory germ cell cancer--a phase I/II trial of high-dose carboplatin and etoposide with autologous bone marrow transplantation. J Clin Oncol..

[20] Lorch A, Kollmannsberger C, Hartmann JT, Metzner B, Schmidt-Wolf IG, Berdel WE (2007). Single versus sequential high-dose chemotherapy in patients with relapsed or refractory germ cell tumors: a prospective randomized multicenter trial of the German Testicular Cancer Study Group. J Clin Oncol..

[21] Einhorn LH, Williams SD, Chamness A, Brames MJ, Perkins SM, Abonour R (2007). High-dose chemotherapy and stem-cell rescue for metastatic germ-cell tumors. N Engl J Med..

[22] Rivera-Franco MM, Leon-Rodriguez E, Castro-Saldaña HL (2017). Costs of hematopoietic stem cell transplantation in a developing country. Int J Hematol..

